# Chemical Tuning Enhances Both Potency Toward Nrf2 and *In Vitro* Therapeutic Index of Triterpenoids

**DOI:** 10.1093/toxsci/kfu080

**Published:** 2014-08-02

**Authors:** Ian M. Copple, Luke M. Shelton, Joanne Walsh, Denise V. Kratschmar, Adam Lister, Alex Odermatt, Christopher E. Goldring, Albena T. Dinkova-Kostova, Tadashi Honda, B. Kevin Park

**Affiliations:** * MRC Centre for Drug Safety Science, Department of Molecular and Clinical Pharmacology, Institute of Translational Medicine, University of Liverpool, Liverpool L69 3GE, UK; † Division of Molecular and Systems Toxicology, Department of Pharmaceutical Sciences, University of Basel, CH-4056 Basel, Switzerland; ‡ Jacqui Wood Cancer Centre, Division of Cancer Research, Medical Research Institute, University of Dundee, Dundee DD1 9SY, UK; § Department of Chemistry and Institute of Chemical Biology & Drug Discovery, Stony Brook University, Stony Brook, New York 11794

**Keywords:** Nrf2, triterpenoids, therapeutic index

## Abstract

The transcription factor Nrf2 protects against a number of experimental pathologies, and is a promising therapeutic target. The clinical investigation of a potent Nrf2-inducing agent, the triterpenoid (TP) bardoxolone methyl (BARD), was recently halted due to adverse cardiovascular events in chronic kidney disease patients, although the underlying mechanisms are yet to be resolved. The majority of small molecule Nrf2 inducers are electrophilic and trigger Nrf2 accumulation via the chemical modification of its redox-sensitive repressor Keap1. Therefore, it is pertinent to question whether the therapeutic targeting of Nrf2 could be hindered in many cases by the inherent reactivity of a small molecule inducer toward unintended cellular targets, a key mechanism of drug toxicity. Using H4IIE-ARE8L hepatoma cells, we have examined the relationship between (a) Nrf2 induction potency, (b) toxicity and (c) *in vitro* therapeutic index (ratio of b:a) for BARD and a number of other small molecule activators of Nrf2. We show that BARD exhibits the highest potency toward Nrf2 and the largest *in vitro* therapeutic index among compounds that have been investigated clinically (namely BARD, sulforaphane and dimethylfumarate). Through further examination of structurally related TPs, we demonstrate that an increase in potency toward Nrf2 is associated with a relatively smaller increase in toxicity, indicating that medicinal chemistry can be used to enhance the specificity of a compound as an inducer of Nrf2 signaling whilst simultaneously increasing its therapeutic index. These findings will inform the continuing design and development of drugs targeting Nrf2.

The transcription factor Nrf2 controls the basal and inducible expression of a battery of genes with diverse physiological roles, including the preservation of redox balance, the metabolism and detoxification of xenobiotics, and the regulation of multiple metabolic pathways that ensure the provision of cellular energy (Ma, 2013). Therefore, Nrf2 plays an important role in the maintenance of homeostasis. This is emphasized by the enhanced susceptibility of transgenic Nrf2 null mice to a number of diseases and chemical toxicities (Copple *et al.*, 2008). Conversely, genetic or pharmacological activation of Nrf2 signaling has shown promise as a strategy for the prevention of a number of pathologies in animal models, prompting interest in the design and development of small molecule inducers of the Nrf2 pathway as novel drug candidates in a variety of clinical contexts (Suzuki *et al.*, 2013). However, it is established that the majority of compounds that stimulate Nrf2 signaling are thiol-reactive (Dinkova-Kostova *et al.*, 2001; Talalay *et al.*, 1988) and capable of modifying cysteine residues in Keap1, the main cytosolic repressor of Nrf2 (Bryan *et al.*, 2013), whilst many existing and withdrawn drugs are known to provoke off-target toxic effects through the generation of electrophilic reactive metabolites (Park *et al.*, 2011). For example, the commonly used analgesic acetaminophen provokes hepatocellular necrosis when taken in overdose, due to the formation of a reactive quinoneimine that depletes glutathione, leading to the covalent modification of numerous macromolecules and the induction of oxidative stress (Hinson *et al.*, 2010). Therefore, it is possible that the pharmacological targeting of Nrf2 could be hindered in many cases by the inherent reactivity of a small molecule inducer toward unintended cellular targets, in addition to Keap1, provoking therapy-limiting toxicity.

Until recently, one of the most promising candidates for a novel therapy based at least partly on the activation of Nrf2 was bardoxolone methyl (BARD). This thiol-reactive triterpenoid (TP) (Couch *et al.*, 2005), known to be one of the most potent small molecule inducers of Nrf2 signaling (Dinkova-Kostova *et al.*, 2005), triggered the improvement of renal function in patients with moderate to severe chronic kidney disease (CKD) and type 2 diabetes (Pergola *et al.*, 2011). However, in 2012, a phase III clinical trial of BARD in patients with severe CKD was prematurely terminated, due to a high incidence of cardiovascular-related adverse events and deaths in the treatment arm, for which the underlying mechanism is currently unknown (de Zeeuw *et al.*, 2013). It has been suggested that such a patient cohort was not the most appropriate for intervention with an Nrf2-targeting therapy, given that stimulation of Nrf2 signaling has been shown to prevent, but not reverse, a number of pathologies in laboratory studies (Zhang, 2013). Moreover, no significant adverse effects were reported in the phase II clinical trial of BARD in patients with diabetic nephropathy (Pergola *et al.*, 2011), or a phase I clinical trial in patients with advanced solid tumors and lymphomas (Hong *et al.*, 2012), despite the drug being administered at relatively high doses for up to a year. Therefore, it is unclear if the cardiovascular events attributed to BARD are intrinsic to the compound, or caused by unintended effects specific to severe CKD patients.

In order to assess whether the medicinal chemistry effort that resulted in the high potency of BARD as an inducer of Nrf2 signaling inadvertently produced a molecule that was intrinsically more toxic to mammalian cells, we have determined its *in vitro* therapeutic index (Fig. 1) alongside the clinically investigated Nrf2 inducers sulforaphane (SUL) and dimethylfumarate (DMF), together with a number of structurally related TPs. Our findings highlight the potential for enhancing the potency of a compound as an Nrf2 inducer whilst simultaneously increasing its therapeutic index.

**FIG. 1.**
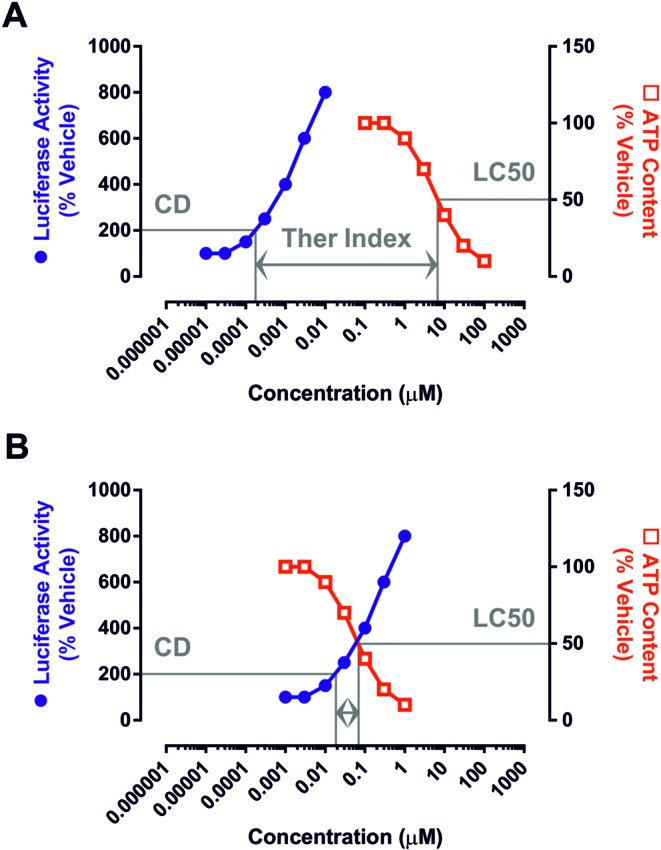
Concept of the *in vitro* therapeutic index. By calculating the concentrations of a compound that provoke a twofold increase in the activity of the Nrf2-sensitive ARE8L reporter transgene (hereafter referred to as the CD value) and a twofold decrease in cellular ATP content (hereafter referred to as the LC50 value) in H4IIE-ARE8L cells, the *in vitro* therapeutic index (ratio of LC50 divided by CD) can be determined. (A) Example of a hypothetical compound exhibiting a relatively large *in vitro* therapeutic index, due to its high potency toward Nrf2 (i.e., low CD value) and minimal toxicity (i.e., high LC50 value). (B) Example of a hypothetical compound exhibiting a relatively small *in vitro* therapeutic index, due to its low potency toward Nrf2 (i.e., high CD value) and high toxicity (i.e., low LC50 value). This concept has been used to examine the relationship between Nrf2 induction potency, toxicity, and *in vitro* therapeutic index for a series of small molecule inducers of Nrf2.

## MATERIALS AND METHODS

### 

#### 

##### Materials

Unless stated, all reagents were obtained from Sigma-Aldrich (Poole, UK).

##### Chemistry

**FIG. 2.**
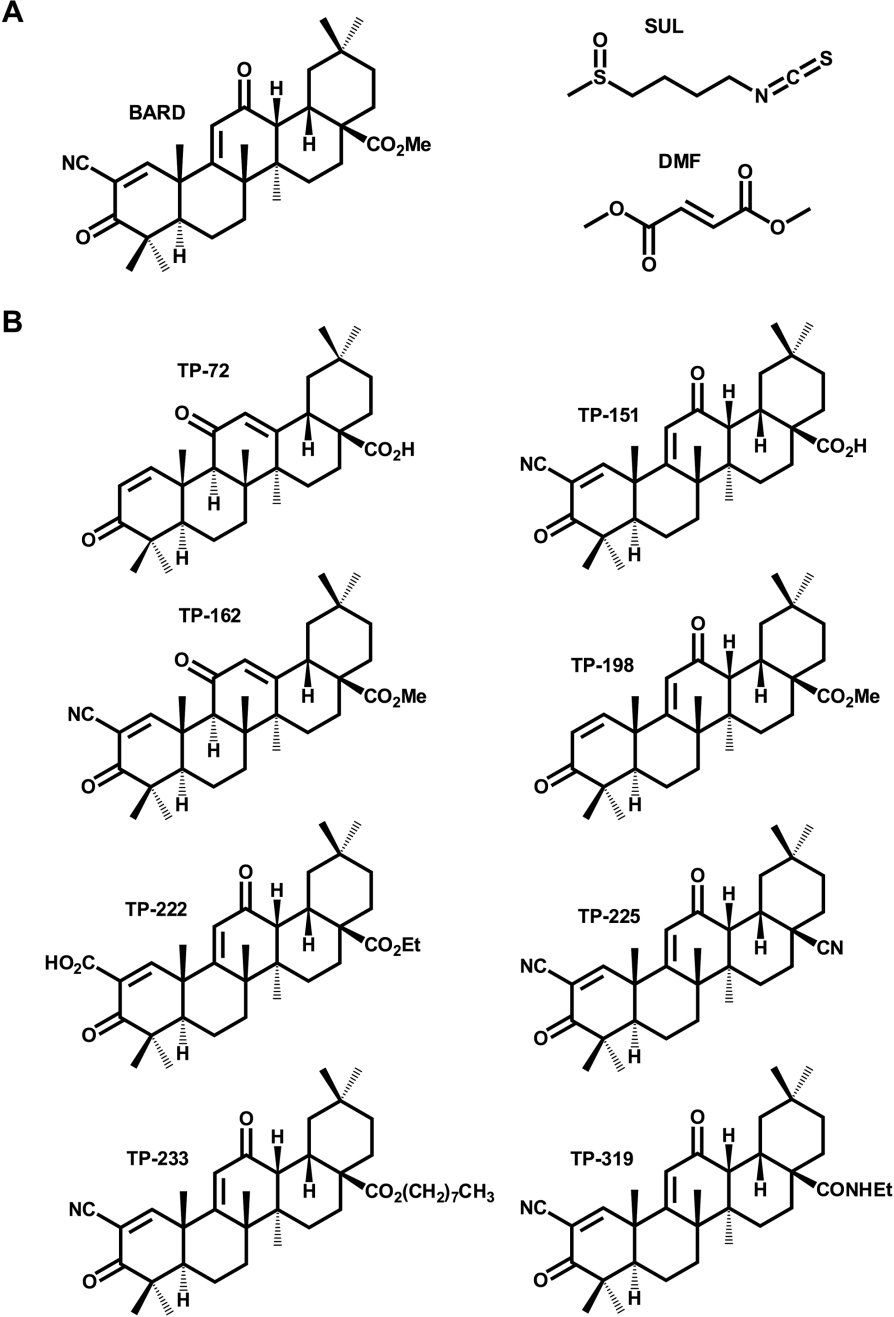
Chemical structures of the Nrf2 inducers used in this study. (A) Chemical structures of BARD, SUL and DMF, Nrf2 inducers that have entered the clinic. (B) Chemical structures of TP analogues of BARD.

BARD and the other TPs were synthesized by the methods described previously (Honda *et al.*, 2000, 2002). Chemical structures of the Nrf2 inducers used in this study are shown in Figure 2.

##### Cell culture

Rat H4IIE-ARE8L cells, stably expressing a luciferase reporter regulated by an eight-times repeated antioxidant response element, were generated as described previously (Kratschmar *et al.*, 2012). Mouse Hepa-1c1c7 and human HepG2 cells were obtained from ATCC. Cells were maintained at 37°C in a 5% CO_2_ atmosphere in Dulbecco's Modified Eagle's Medium supplemented with 584 mg/l l-glutamine, 10% fetal bovine serum (Life Technologies, Paisley, UK). The media for H4IIE-ARE8L cells was further supplemented with 1mM HEPES and 1× nonessential amino acids, whereas the media for Hepa-1c1c7 and HepG2 cells was further supplemented with 100 U/ml penicillin and 100 μg/ml streptomycin. For drug treatments, compounds were dissolved in dimethyl sulfoxide (DMSO), with the concentration of the solvent in the media controlled to 0.5%.

##### Determination of ARE8L reporter activity

Following exposure of H4IIE-ARE8L cells to the indicated compounds for 24 h, reporter assays were performed essentially as described (Kratschmar *et al.*, 2012), using the Bright-Glo Luciferase Assay System (Promega, Southampton, UK), in accordance with the manufacturer's instructions. Data are normalized to the reporter activity detected in vehicle-exposed cells.

##### Determination of cellular ATP content

Following exposure of H4IIE-ARE8L, Hepa-1c1c7 or HepG2 cells to the indicated compounds for 24 h, cellular ATP content was quantified using the CellTiter-Glo Luminescent Cell Viability Assay (Promega), in accordance with the manufacturer's instructions. Data are normalized to the ATP content of vehicle-exposed cells.

##### Calculation of *in vitro* therapeutic index

The concentrations of each compound that provoked a twofold increase in the activity of the ARE8L reporter transgene (CD value) and a twofold decrease in cellular ATP content (LC50 value) in H4IIE-ARE8L cells was determined by nonlinear regression analysis of the respective concentration-response curves, using GraphPad Prism 6 (GraphPad Software). The *in vitro* therapeutic index is expressed as a ratio of LC50 divided by CD.

##### Data analysis

Data are presented as the mean ± SD of three independent experiments. Pearson correlation coefficients were determined using GraphPad Prism 6.

## RESULTS

### 

#### BARD has a Large In Vitro Therapeutic Index Relative to other Clinically Validated Nrf2 Inducers

**FIG. 3.**
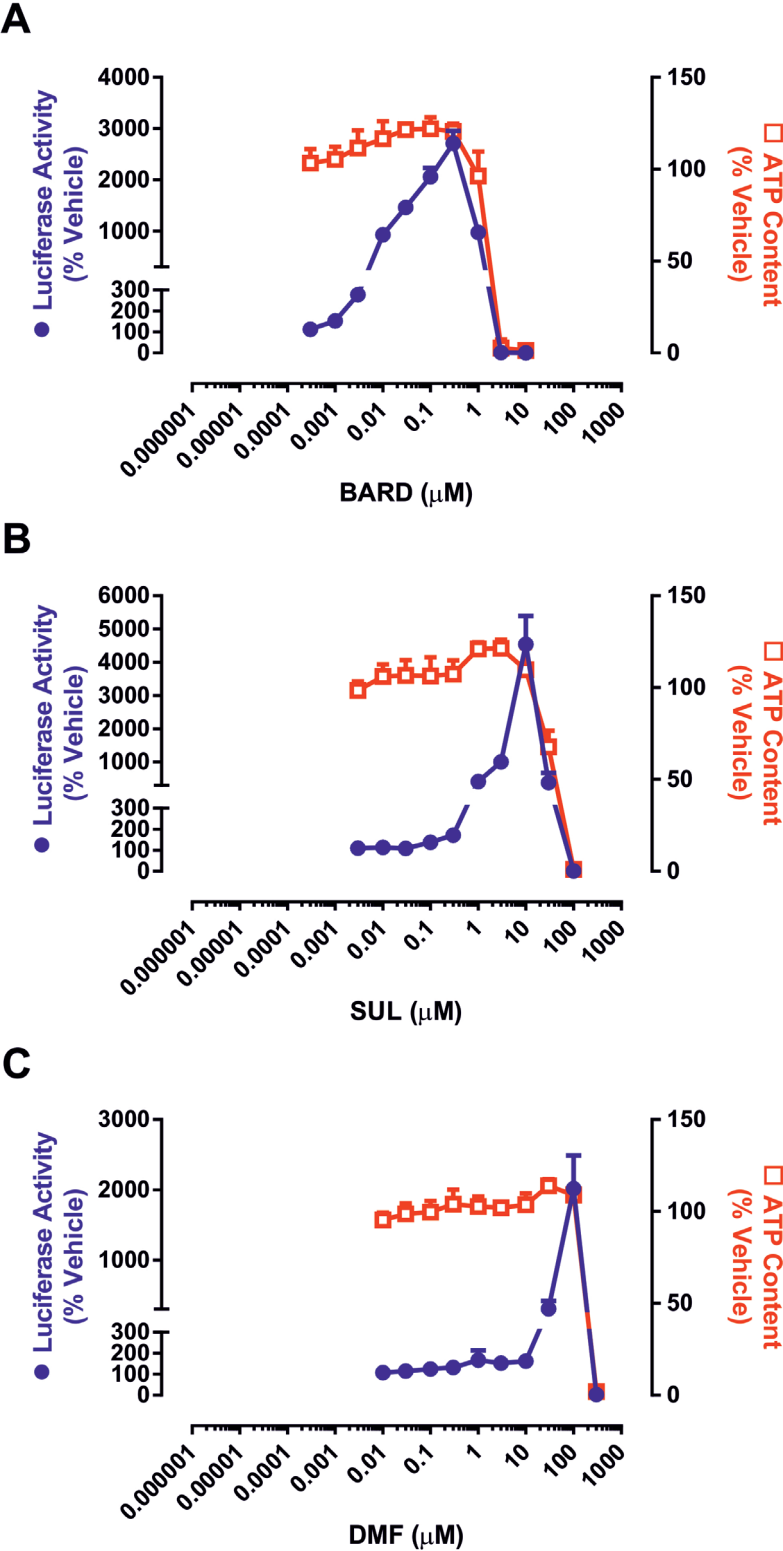
Pharmacological potencies and toxicities of clinically validated Nrf2 inducers in H4IIE-ARE8L cells. Cells were exposed to the indicated concentrations of (A) BARD, (B) SUL, or (C) DMF for 24 h. Luciferase reporter activity (circles) and ATP content (squares) were subsequently quantified as readouts of Nrf2 induction and toxicity, respectively. Data represent mean + SD, *n* = 3.

We first examined the relationship between the potency of BARD as an inducer of Nrf2 signaling and its ability to provoke cell death alongside SUL and DMF (Fig. 2), Nrf2 activators which have recently undergone patient trials in different disease contexts (Houghton *et al.*, 2013; Lee *et al.*, 2013). In order to enable a comparison of Nrf2 induction potency and toxicity within the same experimental setting, we used rat hepatoma H4IIE-ARE8L cells, which stably express an Nrf2-responsive luciferase transgene (Kratschmar *et al.*, 2012). All three compounds were able to enhance the activity of the Nrf2 reporter transgene, in a concentration-dependent manner, in H4IIE-ARE8L cells (Fig. 3). Calculation of the CD values revealed a rank order of potency of BARD > SUL > DMF (Table 1). At higher concentrations, all three compounds triggered a loss of cell viability in H4IIE-ARE8L cells, concomitant with a loss of Nrf2 reporter transgene activity (Fig. 3), the latter likely due to the disruption of critical cellular process and/or a lack of ATP necessary for the generation of bioluminescence by luciferase. Calculation of the LC50 values also revealed a rank order of potency of BARD > SUL > DMF (Table 1). Similar LC50 values were obtained from parallel experiments performed with mouse Hepa-1c1c7 and human HepG2 hepatoma cells, indicating species conservation (Supplementary Data). We noted that, although the three compounds exhibited CD values spanning four orders of magnitude, their respective LC50 values differed by a maximum of two orders of magnitude. That an increase in potency toward Nrf2 is associated with a relatively smaller increase in toxicity was verified by calculation of the *in vitro* therapeutic index for each compound (Table 1), which again revealed a rank order of BARD > SUL > DMF, confirming that BARD has by far the largest *in vitro* therapeutic index of Nrf2 inducers that have entered the clinic to date, and indicating that an increase in potency toward Nrf2 results in a relative enhancement of *in vitro* safety (Fig. 5).

**FIG. 4.**
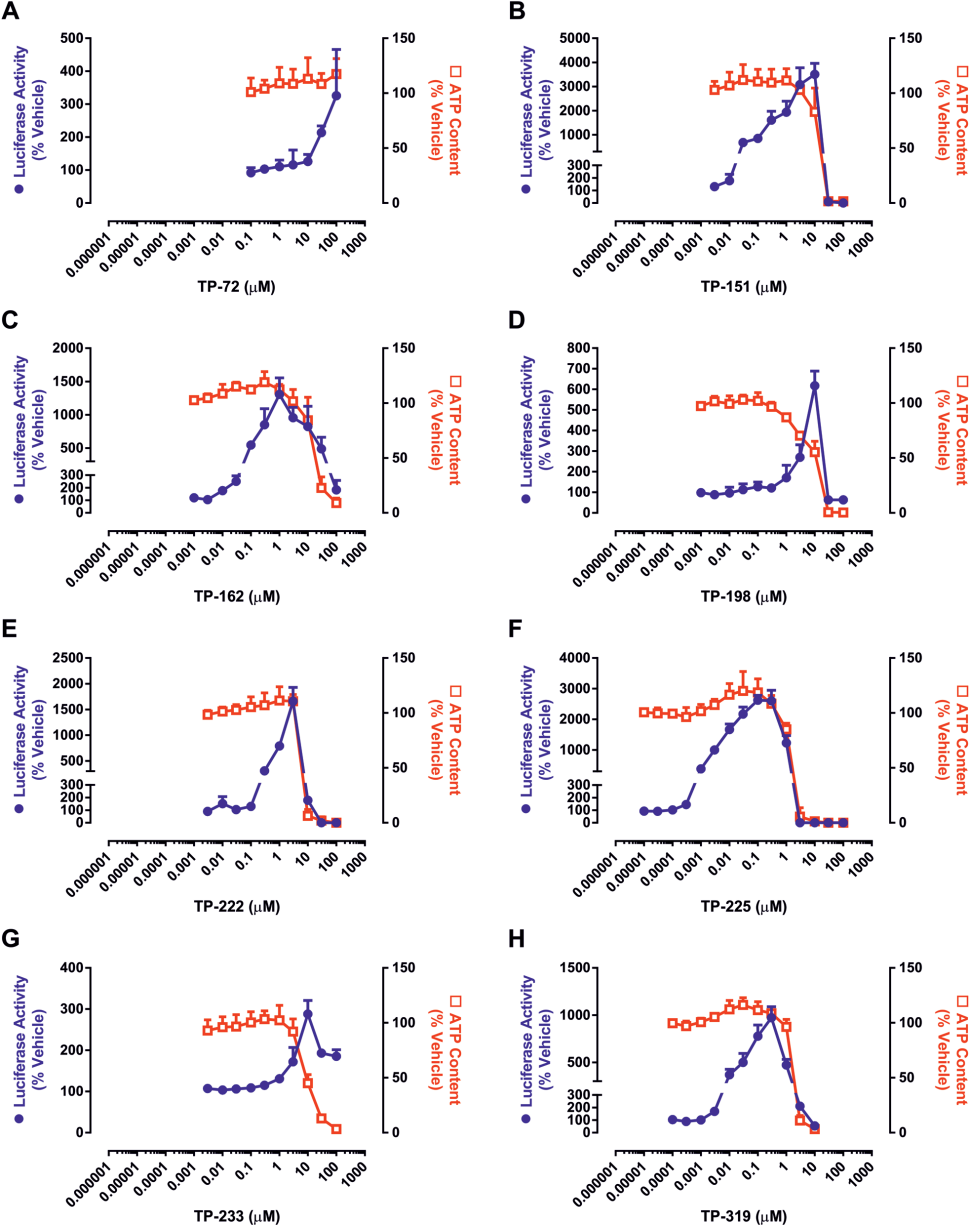
Pharmacological potencies and toxicities of triterpenoids in H4IIE-ARE8L cells. Cells were exposed to the indicated concentrations of (A) TP-72, (B) TP-151, (C) TP-162, (D) TP-198, (E) TP-222, (F) TP-225, (G) TP-233, or (H) TP-319 for 24 h. Luciferase reporter activity (circles) and ATP content (squares) were subsequently quantified as readouts of Nrf2 induction and toxicity, respectively. Data represent mean + SD, *n* = 3.

**FIG. 5.**
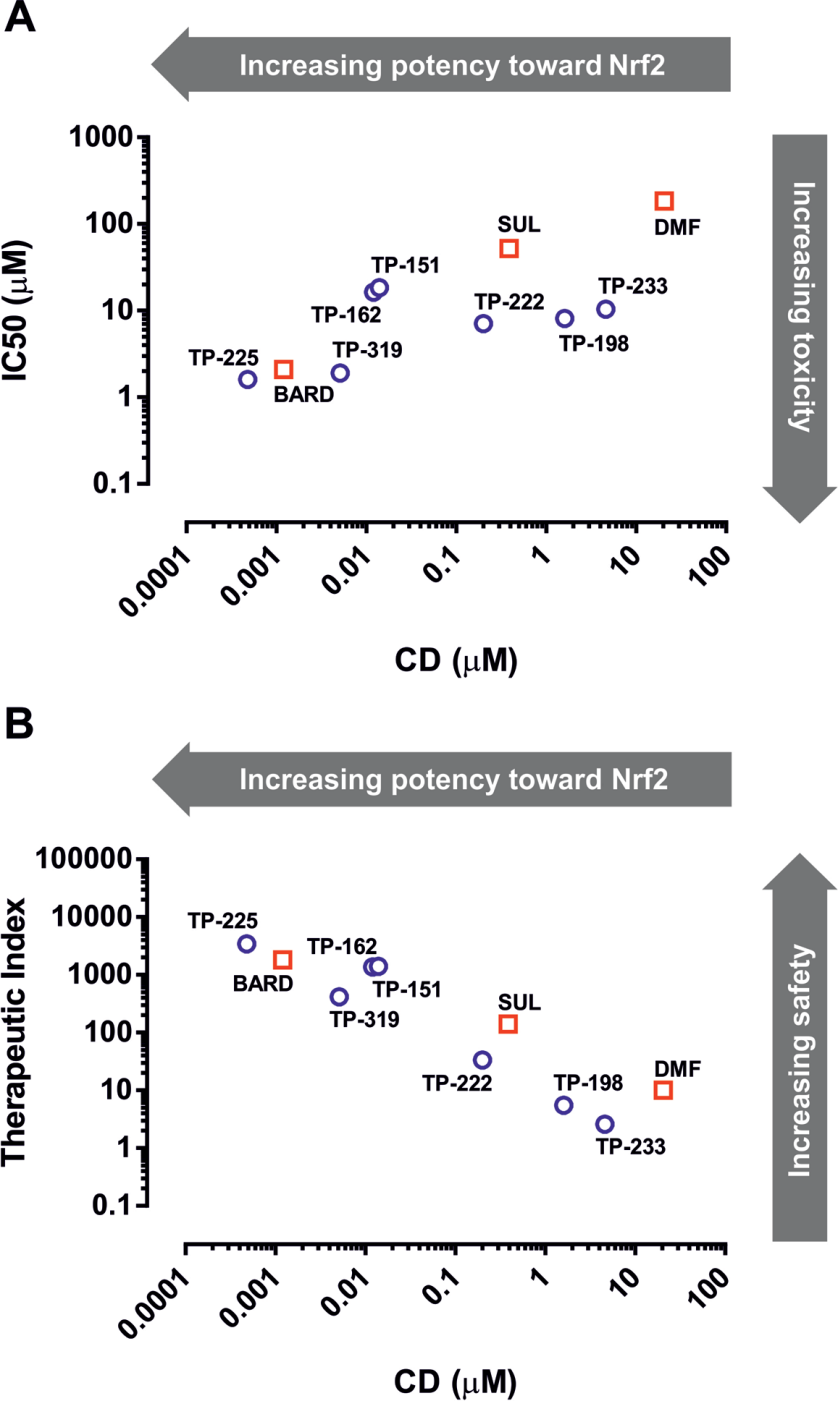
Correlation between potency toward Nrf2, toxicity and *in vitro* therapeutic index of Nrf2 inducers in H4IIE-ARE8L cells. Correlation between potency toward Nrf2 and (A) toxicity [Pearson correlation coefficient 0.74, *p* = 0.014] or (B) *in vitro* therapeutic index [Pearson correlation coefficient −0.925, *p* = 0.0001] of clinically validated Nrf2 inducers (squares) and TPs (circles). CD and LC50 values and therapeutic index for each compound were calculated as described.

**TABLE 1:** Pharmacological Potencies, Toxicities and *In Vitro* Therapeutic Indices of Nrf2 Inducers in H4IIE-ARE8L Cells

	CD*^a^* (μM)	LC50*^b^* (μM)	Therapeutic index*^c^*
TP-225	0.0005 ± 0.0001	1.6 ± 0.2	3448.3 ± 698.2
BARD	0.001 ± 0.0001	2.1 ± 0.5	1818.1 ± 356.3
TP-162	0.02 ± 0.009	18.4 ± 4.6	1397.7 ± 415.1
TP-151	0.01 ± 0.004	16.3 ± 3.7	1373.3 ± 201.5
TP-319	0.005 ± 0.001	1.9 ± 0.2	417.7 ± 72.1
SUL	0.4 ± 0.1	51.9 ± 2.6	140.6 ± 38.4
TP-222	0.2 ± 0.02	7.2 ± 0.3	33.5 ± 2.1
DMF	20.5 ± 8.7	184.4 ± 4.8	10.0 ± 3.7
TP-198	1.6 ± 0.6	8.1 ± 1.4	5.5 ± 1.4
TP-233	4.6 ± 2.1	10.4 ± 2.6	2.6 ± 1.4
TP-72	29.1 ± 2.7	N.D.*^d^*	N.D.*^d^*

*^a^*Concentration that provokes a twofold increase in ARE8L reporter transgene activity.

*^b^*Concentration that provokes a twofold decrease in cellular ATP content.

*^c^*Ratio of LC50 divided by CD.

*^d^*Not determined, due to lack of ATP depletion at the highest possible concentration (100μM).

#### Chemical Tuning of TPs Enhances Nrf2 Induction Potency and In Vitro Therapeutic Index

To provide a chemical insight into the relationship between Nrf2 induction potency and toxicity of BARD, we expanded our analyses to include structurally related TPs (Fig. 2) which were previously shown to possess a range of chemical reactivities that correlated with potency as inducers of Nrf2 signaling (Bensasson *et al.*, 2010). In keeping with this, we found that TP-72, TP-151, TP-162, TP-198, TP-222, TP-225, TP-233, and TP-319 evoked concentration-dependent increases in Nrf2 reporter transgene activity in H4IIE-ARE8L cells (Fig. 4), with CD values spanning four orders of magnitude (Table 1). Importantly, these CD values and the resulting rank order of potency toward Nrf2 were in excellent agreement with our previous structure-activity relationship (SAR) study of these compounds as inducers of NAD(P)H:quinone oxidoreductase 1 activity in Hepa-1c1c7 cells (Dinkova-Kostova *et al.*, 2005), demonstrating the robustness of our analytical methods. With the exception of TP-72 (see below), the TPs induced loss of cell viability at micromolar concentrations (Fig. 4), with LC50 values spanning one order of magnitude (Table 1).

In keeping with the above observations, calculation of the *in vitro* therapeutic index for each TP confirmed that an increase in potency toward Nrf2 is associated with a relative enhancement of *in vitro* safety (Fig. 5, Table 1). Specifically, TP-151, TP-162, TP-225, and TP-319, which similarly to BARD contain an electron-withdrawing nitrile group (that increases the Michael reactivity) at C-2 on ring A, exhibited *in vitro* therapeutic indices 10–100 times greater than TP-222, which has a carboxyl group at this position (Table 1). Consistent with this, TP-198, which lacks the nitrile group and only contains an enone function on this ring, was shown to have an *in vitro* therapeutic index 300 times lower than BARD (Table 1). In addition, a second nitrile group at C-17 was shown to further increase the *in vitro* therapeutic index by 2–3 times (compare TP-225 with BARD and TP-151; Table 1). In contrast, a long side chain substitution (nine carbons) at the same position lowered the *in vitro* therapeutic index substantially; TP-233 was one of the least potent Nrf2 inducers and had the lowest *in vitro* therapeutic index among the TPs tested (Table 1). Although it was not possible to calculate an LC50 value and, therefore, *in vitro* therapeutic index for TP-72, because it did not provoke loss of cellular ATP content at the highest possible concentration of 100μM (Fig. 4), this compound exemplified the combined importance of the nitrile group on ring A and the “correct” position of the enone on ring C for potency toward Nrf2. Indeed, TP-72, which lacks both of these functions, was found to be 30,000 times less potent than BARD as an inducer of Nrf2 (Table 1). Taken together, these data confirm the favorable association between Nrf2 induction potency and *in vitro* therapeutic index for TPs and other small molecule Nrf2 inducers.

## DISCUSSION

The delay between the decision of the Independent Data Monitoring Committee to stop the phase III clinical trial of BARD in patients with severe CKD “for safety concerns due to excess serious adverse events and mortality” and the recent publication of the study data (de Zeeuw *et al.*, 2013) provided opportunity for both speculation surrounding the exact nature of the adverse events and commentary on the implications of the trial failure for targeting Nrf2 in the clinic (Rossing, 2013; Tayek and Kalantar-Zadeh, 2013; Zhang, 2013). In light of the well-established chemical properties (i.e., electrophilicity and reactivity with protein thiols) of the majority of Nrf2 inducers, it was pertinent to question whether the therapeutic targeting of Nrf2 could be undermined by the inherent reactivity of a small molecule inducer toward unintended cellular targets, a key mechanism of drug toxicity (Park *et al.*, 2011). Here, we have examined this concept from a chemical perspective, in a model cell system, by defining the relationship between the potency of a compound as an inducer of Nrf2 and its *in vitro* therapeutic index, for molecules that have entered the clinic (BARD, SUL, and DMF) and a representative selection of TPs.

The principal finding of this study is that medicinal chemistry can be used to enhance the potency of a compound toward Nrf2 without adversely affecting its *in vitro* therapeutic index. Indeed, for the compounds studied here, we have demonstrated that an increase in Nrf2 induction potency across four orders of magnitude is accompanied by an increase in toxicity across just two orders of magnitude (Fig. 5A), and therefore an effective overall enhancement of *in vitro* safety (Fig. 5B). The fact that these conclusions apply to a series of structurally related compounds (BARD and the other TPs) as well as structurally distinct molecules (SUL and DMF) implies that our findings are likely applicable to a range of Nrf2 inducers, and indicate that Nrf2 potency is determined not by universal reactivity toward protein thiols, but via specific reactivity with a critical target, likely Keap1. Indeed, SUL and DMF (or, more precisely, its major intracellular metabolite monomethylfumarate) have been shown to directly modify specific cysteine residues in Keap1 (Hu *et al.*, 2011; Linker *et al.*, 2011), and we have previously demonstrated that TP-225 can interact with purified recombinant Keap1 *in vitro* (Dinkova-Kostova *et al.*, 2005). Therefore, the identification of potent Nrf2 inducers may be supported by the *in silico* or *in vitro* screening of compound reactivity toward Keap1, as described recently (Hu *et al.*, 2012; Wu *et al.*, 2010), although *in silico* approaches would be considerably aided by the resolution of the full-length crystal structure of Keap1.

Notably, TP-225, and not BARD, exhibited the largest *in vitro* therapeutic index of the TPs (and indeed all of the compounds) studied here. Consistent with our contention that potency toward Nrf2 is the major contributory factor in the determination of *in vitro* safety, to our knowledge TP-225 is the most potent inducer of Nrf2 reported to date (Dinkova-Kostova *et al.*, 2005). We have previously demonstrated the chemopreventive efficacy of TP-225 in SKH-1 hairless mice exposed to ultraviolet B radiation, in which topical application of the compound was shown to stimulate Nrf2 signaling and reduce the appearance of premalignant lesions and the volume of malignant tumors (Dinkova-Kostova *et al.*, 2008). Despite these promising properties, the development of TP-225 as a therapeutic agent has been hindered by its poor oral bioavailability (unpublished data).

Determination of the *in vitro* therapeutic index provides an informative means of comparing the relationship between Nrf2 induction potency and toxicity for a number of compounds within a stable cell system. However, it cannot indicate with any degree of certainty that a compound will or will not provoke adverse reactions in animals and humans. Many clinical drug toxicities are underpinned by complex mechanisms involving species-specific drug disposition, interactions between different cell types, or unexpected biological perturbations in specific disease cohorts. Therefore, the balance between pharmacological potency, therapeutic efficacy, and toxicity is likely to be considerably different *in vivo* than in our *in vitro* system. Indeed, although DMF exhibited one of the lowest *in vitro* therapeutic indices here, this compound has recently been licensed for the management of multiple sclerosis (Lee *et al.*, 2013), whereas BARD has recently been shown to provoke adverse cardiovascular events in CKD patients (de Zeeuw *et al.*, 2013), despite it demonstrating one of the highest *in vitro* therapeutic indices in this study. Although these observations could indicate that high Nrf2 induction potency/Keap1 specificity is associated with worse clinical outcome and/or increased risk of adverse events in patients, two factors argue against this. Firstly, the relatively limited clinical experience of using Nrf2 inducers (BARD, SUL, and DMF) in different disease contexts (CKD, cancer, and multiple sclerosis) makes it difficult, at this stage, to draw conclusions about the precise relationship between Nrf2 induction potency and clinical efficacy for a given compound. Secondly, the lack of significant toxicity, particularly of a cardiovascular nature, in a recent year-long phase I clinical trial of BARD in cancer patients (Hong *et al.*, 2012) suggests that the adverse reactions observed in CKD patients are driven by unforeseen effects of the drug in this specific patient cohort (which are known to be predisposed to cardiovascular disease), rather than via chronic activation of Nrf2 *per se*. This contention is further supported by the recent commencement of a phase II clinical trial of BARD in patients with pulmonary arterial hypertension (NCT02036970).

TPs are known to target numerous biological processes (Liby and Sporn, 2012), and it has yet to be proven, to our knowledge, that induction of Nrf2 has a direct mechanistic role in the clinical actions of BARD in CKD patients, although Nrf2 has been shown to be protective in animal models of renal disease (Ruiz *et al.*, 2013; Shelton *et al.*, 2013). An understanding of whether BARD provokes cardiovascular dysfunction in CKD patients by direct (e.g., cytotoxicity toward a particular cell type) or indirect (e.g., via hypomagnesemia or blood pressure perturbation) means will inform detailed mechanistic studies in relevant model systems, which could further distinguish undesirable compound-patient interactions from generic deleterious effects. Such studies will provide insights into the human biological factors that modulate the efficacy and safety of Nrf2 inducers *in vivo*. This in turn could lead to the development of more sophisticated model cell systems that could be used in the early development of novel Nrf2 inducers for specific clinical indications and target patient populations.

## SUPPLEMENTARY DATA

Supplementary data are available online at http://toxsci.oxfordjournals.org/.

## FUNDING

Medical Research Council as part of the Centre for Drug Safety Science (G0700654); European Community Innovative Medicines Initiative as part of the MIP-DILI project (115336); Cancer Research UK (C20953/A10270); Reata Pharmaceuticals.

## Supplementary Material

Supplementary Data
