# Supplementary material for: Chemical Tuning Enhances Both Potency Toward Nrf2 and In Vitro Therapeutic Index of Triterpenoids
Source: Toxicol Sci. 2014 Aug 2;140(2):462–9. doi: 10.1093/toxsci/kfu080 (PMC4120102; doi:10.1093/toxsci/kfu080)
Supplement: Supplementary Data [file supp_140_2_462__index.html]

Chemical Tuning Enhances Both Potency Toward Nrf2 and In Vitro Therapeutic Index of Triterpenoids — Chemical Tuning Enhances Both Potency Toward Nrf2 and In Vitro Therapeutic Index of Triterpenoids — Chemical Tuning Enhances Both Potency Toward Nrf2 and In Vitro Therapeutic Index of Triterpenoids — Supplementary Data 

# Chemical Tuning Enhances Both Potency Toward Nrf2 and *In Vitro* Therapeutic Index of Triterpenoids

## Supplementary Data

**Files in this Data Supplement:**

- Supplementary Data
