# Supplementary material for: Chemical Tuning Enhances Both Potency Toward Nrf2 and In Vitro Therapeutic Index of Triterpenoids
Source: Toxicol Sci. 2014 Aug 2;140(2):462–9. doi: 10.1093/toxsci/kfu080 (PMC4120102; doi:10.1093/toxsci/kfu080)
Supplement: Supplementary Data [file supp_kfu080_toxsci-14-0086-File007.docx]

**Chemical Tuning Enhances both Potency Towards Nrf2 and In Vitro Therapeutic Index of Triterpenoids**

Ian M. Copple, Luke M. Shelton, Joanne Walsh, Denise V. Kratschmar, Adam Lister, Alex Odermatt, Christopher E. Goldring, Albena T. Dinkova-Kostova, Tadashi Honda, B. Kevin Park.

**SUPPLEMENTARY DATA**

Fig. S1; Toxicity of Nrf2 inducers in H4IIE-ARE8L, Hepa-1c1c7 and HepG2 cells.

Fig. S2; Comparison of toxicities of Nrf2 inducers in H4IIE-ARE8L, Hepa-1c1c7 and HepG2 cells.

**Fig. S1; Toxicity of Nrf2 inducers in H4IIE-ARE8L, Hepa-1c1c7 and HepG2 cells.** Cells were exposed to the indicated concentrations of each compound for 24 h. ATP content was subsequently quantified as a readout of cell viability. Data are expressed relative to the ATP content of cells exposed only to vehicle (DMSO, 0.5%). Data represent mean + SD, n=3.

**Fig. S2; Comparison of toxicities of Nrf2 inducers in H4IIE-ARE8L, Hepa-1c1c7 and HepG2 cells.** LC50 values for each compound were calculated by non-linear regression analysis of the respective concentration-response curves depicted in Fig. S1. A visual comparison of the rank order of toxicity of the compounds reveals consistency across the three cell lines, indicating species conservation.
